# Quantitative proteomics analysis of papillary thyroid carcinoma reveals protein S, clusterin, and leucine-rich α-2-glycoprotein 1 as potential prognostic protein biomarkers

**DOI:** 10.1097/MD.0000000000043715

**Published:** 2025-08-08

**Authors:** Haili Sun, Jianbiao Wang, Nizhen Xu, Kehao Le

**Affiliations:** a Nursing Department, the Affiliated Sir Run Run Shaw Hospital, Zhejiang University School of Medicine, Hangzhou, Zhejiang, P. R. China; b Department of Head and Neck Surgery, the Affiliated Sir Run Run Shaw Hospital, Zhejiang University School of Medicine, Hangzhou, Zhejiang, P. R. China.

**Keywords:** CLU, LRG1, papillary thyroid carcinoma, prognosis biomarkers, PROS1, quantitative proteomics

## Abstract

Papillary thyroid carcinoma (PTC) is the most common type of thyroid cancer. The primary challenge is identifying patient subgroups with PTC and choosing the most effective treatment approach. To explore the differentially expressed proteins (DEPs) between high and low recurrent-risk PTCs, we collected 15 tissues comprising high (n = 7) and low (n = 8) recurrent-risk groups from PTC. The samples were detected by tandem mass tag labeling proteomics. Using The Cancer Genome Atlas data on thyroid cancer, prognosis-related DEPs were identified. Furthermore, an immunohistochemistry stain of 53 cases of PTC tumors was adopted to validate the relation of potential biomarkers with prognosis. We identified 8958 proteins from the 15 samples, with 95 DEPs obtained by comparing high and low recurrent-risk groups, including 38 upregulated and 57 downregulated proteins. Three downregulated proteins (protein S [PROS1], clusterin [CLU], and leucine-rich α-2-glycoprotein 1 [LRG1]) were found to be significantly associated with poor overall survival in thyroid cancer using differential analysis and Kaplan–Meier survival analysis. Immunohistochemistry results showed low or moderated expressions of PROS1, CLU, and LRG1 were significantly associated with high-risk clinicopathologic characteristics of PTC. PTC patients with higher expression of PROS1, CLU, and LRG1 had better progression-free survival than those with low or moderate expression. Our study identified PROS1, CLU, and LRG1 as novel prognostic biomarkers in PTC.

## 1. Introduction

Thyroid cancer represents the most frequent endocrine malignancy and its incidence has increased significantly in recent decades.^[1–3]^ Moreover, the majority of thyroid carcinomas arise from thyroid follicular cells, and the ratio can be divided into 2 histological types: papillary thyroid carcinoma (PTC) (80%–85% of cases) and follicular thyroid carcinoma (10%–15% of cases), respectively, commonly referred to as differentiated thyroid carcinomas (DTC).^[4]^ Although patients with DTCs frequently receive thyroidectomy, radioactive iodine (RAI) therapy, and thyroid-stimulating hormone suppression therapy, local recurrence and distant metastasis invariably occur up to 20% and 10%, respectively.^[5]^ Two-thirds of these patients developed RAI-refractory DTC.^[5,6]^ The 10-year survival rate for patients with DTC exhibiting RAI refractoriness is <10%.^[6]^

Unfortunately, no clinical features^[7]^ can reliably distinguish the relatively small number of PTC patients who will develop clinically significant progression from the larger population of people who have indolent PTCs that will not cause significant disease. Prognostic biomarkers are thus required to aid in the early detection of advanced PTC.

Because it allows for the simultaneous detection of thousands of proteins, mass spectrometry-based proteomics is now regarded as a powerful screening tool for identifying differential protein expression patterns.^[8]^ In comparison to studies of the genome and transcriptome, research into the thyroid cancer proteome is in its early stages. Finding differentially expressed proteins (DEPs) between benign and malignant thyroid cancers was the focus of earlier proteomic research.^[9]^ However, there have not been any published proteomic studies on PTC biomarkers for poor prognosis. Since proteins are ultimately the functional effectors of biological activity in cancer cells, we suggested that identifying potential prognosis biomarkers in PTC may be particularly sensitive with global proteomic analysis.

In this study, we compared the global proteomic profiling of PTC tissues based on recurrent-risk stratification. The DEPs in high recurrent-risk PTC tissues compared with low recurrent-risk tissues were identified, then analyzed in bioinformatics, and finally validated through an immunohistochemistry (IHC) study. According to our knowledge, this is the first study to use tandem mass tag labeling proteomics to identify DEPs between PTC tissues with high and low recurrent risk.

## 2. Materials and methods

### 2.1. PTC tissue sample preparation and proteomic data analysis

Fifteen formalin-fixed paraffin-embedded (FFPE) PTC tissues were taken from each patient and organized according to the pathology-identified tumorous area. All procedures involving human material have been approved by the ethics committee of the Sir Run Run Shaw Hospital, School of Medicine, Zhejiang University, and each PTC patient has signed an informed consent form. Previous accounts^[10,11]^ followed the methodology we employed; 0.5 to 1.0 mg of FFPE tissue was cut and processed for each sample. The samples were dewaxed in heptane for 2-time 10 minutes (Sigma-Aldrich LLC, St. Louis) and successively in 100% ethanol (Sigma-Aldrich LLC), 90% ethanol, and 75% ethanol at room temperature each for 5.0 minutes. Then 0.1% formic acid (Thermo Fisher Scientific, St. Louis) was added and shaked for 30.0 minutes, and then the reaction buffer in pH 10.0 condition was exchanged and incubated in a condition of 100 mM Tris-HCl (pH 10.0, Sigma-Aldrich LLC) at 95.0°C for 30.0 minutes. Dewax samples were lysed, according to the previously published paper, in 6.0 M urea (Sigma-Aldrich LLC), 2 M thioureas (Sigma-Aldrich LLC), 10 mM tris-(2-carboxyethyl)phosphine (Sigma-Aldrich LLC), and 40 mM iodoacetamide (Sigma-Aldrich LLC) assisted by pressure cycling technology (PCT) programmed for 90 cycles of 30 seconds at 45,000 psi and 10 seconds at ambient pressure with the temperature of 30°C. PCT assisted digestion using lysC (Hualishi Tech. Ltd, Beijing, China) at a ratio of 1:80 to protein and trypsin (Hualishi Tech. Ltd) at a ratio of 1:20 to protein. PCT was programmed for 120 cycles of 50 seconds at 20,000 psi and 10 seconds at ambient pressure at 30°C. Then, peptides were desalted by C18 before tandem mass tag labeling. The peptide concentration was determined using Nanoscan (Analytik Jena AG, Jena, Germany), and TMT10plex Isobaric Label Reagent (Thermo Fisher Scientific, Rockford) was used to label 20 μg of peptides from each sample as directed. The reaction was stopped with 5% hydroxylamine after labeling, and each batch of 10 samples was combined into one tube and cleaned with a 50 mg C18 column (Sep-Pak Vac, Waters Corporation, Milford).

High-pH fractionation was performed with 120 minutes gradient using XBridge BEH130 C18 Peptide Separation Technology column (130 Å, 3.5 µm, 4.6 mm × 250 mm, 1/pk; Waters Corporation) on Thermo Dinex Ultimate 3000 (Thermo Fisher Scientific, San Jose). The gradient was from 5% to 32% acetonitrile with a flow rate of 1 mL/min, pH 10.0, and 120 fractions were gathered and then evenly divided into 30 fractions.

Each fraction was divided into Thermo Dinex Ultimate 3000 HPLC and analyzed on Q Exactive HF Hybrid Orbitrap (Thermo Fisher Scientific, San Jose) in the mode of data-dependent acquisition (DDA) with 60 minutes effective gradient from 3% to 28% buffer B (98% acetonitrile in water with 0.1% formic acid) at a flow rate of 300 nL/min. The mass spectrometry settings are the same as our previous published literature.^[12]^ A FASTA file containing 20,367 entities from the Swiss-Prot HUMAN database, downloaded on January 22, 2020, was used to search DDA raw data against Protein Discoverer 2.2.0.388. For all searches, fragment mass tolerance was 0.02 Da, and precursor mass tolerance was 10 ppm. The result was adjusted to a strict false discovery rate of 1% at the peptide spectrum match level.

### 2.2. Bioinformatics analysis of DEPs

Using R, the DEPs between high and low recurrent-risk groups were analyzed, and the volcano figure was created with the filters fold change >1.2 and adjusted *P* value < .05. The TCGA (The Cancer Genome Atlas) provided the clinical details and whole transcriptome sequencing data for thyroid cancer. The median expression level was taken as the limit to divide the data into a high-expression group and low-expression group. STRING (https://cn.string-db.org/) was used to study the relationship of differential proteins, and “full STRING network,” “evidence,” and “medium confidence (0.400)” was selected for basic settings. Gene Ontology (GO) and Kyoto Encyclopedia of Genes and Genomes (KEGG) pathway functional enrichment analyses were conducted through the “cluster profiler” R package to assign various biological processes, molecular functions, cellular components as well as pathways of DEPs in the interesting cluster; *P* < .05 was regarded as statistically enriched. A Kaplan–Meier survival analysis was then performed. Using Link Finder modules in Linked Omics, the differentially expressed genes connected to the corresponding protein in the TCGA thyroid cancer data were analyzed (http://www.linkedomics.org/login.php). The Pearson correlation coefficient was selected for statistical analysis. Using the Link Interpreter module, pathway and network analyses of differentially expressed genes were carried out. A false discovery rate of <0.05 was required as the rank criterion for overrepresentation enrichment analysis, which was applied. Gene set enrichment analysis was conducted using the thyroid cancer dataset to understand the thyroid cancer biological pathway.

### 2.3. IHC and evaluation of staining

A set of 53 paraffin-embedded archival PTC specimens, collected between January 2006 and December 2020, was obtained from the Department of Pathology, The Affiliated Sir Run Run Shaw Hospital, School of Medicine; each PTC patient has signed an informed consent form, and Zhejiang University has approved all procedures involving human material. The steps that followed involved performing immunohistochemical staining. The slides were deparaffinized in xylene for 5 minutes 2 times and rehydrated through a graded series of ethanol concentrations (in 100%, 100%, 96%, 96%, and 70% ethanol for 5 minutes, respectively). Antigen retrieval was performed in 98°C water for 20 minutes with 0.01 M citrate buffer. The sections were incubated with 3% hydrogen peroxide for 20 minutes at room temperature and blocked with 10% BSA for 1 hour at room temperature. First antibody (protein S [PROS1] antibody [1:500], clusterin [CLU] antibody [1:50], and leucine-rich α-2-glycoprotein 1 [LRG1] antibody [1:200] [Abcam, Biotechnology, Cambridge, UK]; diluted in 1% BSA-PBS [add only 1% BSA-PBS to controls]) was incubated overnight at 4°C. At room temperature, the anti-rabbit secondary antibody was incubated for 30 minutes. The slides were visualized using 3,3′-diaminobenzidine substrate liquid (Thermo Scientific, St. Louis) and washed with deionized water before hematoxylin counterstaining.

Two researchers blindly assessed the immunostaining to reach a consensus on the staining patterns seen under a light microscope. To evaluate the levels of protein expression, a quantitative score was created by combining the staining area and intensity scores for each case. The first step was to calculate a quantitative score by estimating the proportion of immune-positive cells: 0, no staining of cells in any microscopic fields; 1+, <30% of tissue stained positive; 2+, between 30% and 60% stained positive; and 3+, >60% stained positive. Second, the intensity of staining was scored by evaluating the average staining intensity of the positive cells (0, no staining; 1+, mild staining; 2+, moderate staining; 3+, intense staining). Following that, a final score was created by combining the area score and intensity score for each case (ranging from 0 to 6). A combined staining score of ≤2 was regarded as negative staining (no expression), a score between 3 and 4 as moderate staining (expression), and a score between 5 and 6 as strong staining (high expression).

### 2.4. Statistical analysis

**Figure 1.**
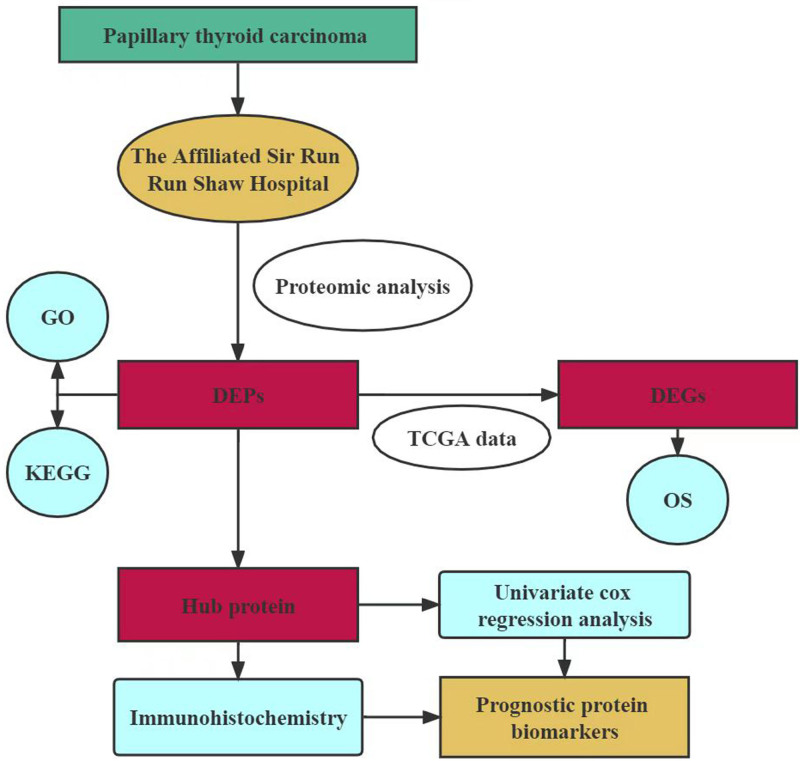
Schematic representation of the experimental workflow of the study. DEG = differentially expressed gene, DEP = differentially expressed protein, GO = Gene Ontology, KEGG = Kyoto Encyclopedia of Genes and Genomes, OS = overall survival, TCGA = The Cancer Genome Atlas.

The Pearson *χ*^2^ test was used to compare group differences. The Mann–Whitney *U* test was used to examine the relationships between the levels of expression of candidate prognostic biomarkers and various clinicopathological characteristics. The Kaplan–Meier survival analysis was used to calculate overall survival (OS) and cumulative progression-free survival (PFS), and to compare the variations, the log-rank method was employed. Two-tailed *P* < .05 was considered as statistical significance. Statistical analysis software was R 3.5.3 (R Foundation for Statistical Computing, Vienna, Austria), GraphPad Prism 8 (GraphPad Software, San Diego), and SPSS version 16.0 (IBM Corporation, Armonk). Figure 1 shows the study flow diagram.

## 3. Results

### 3.1. Global protein profiles of high and low recurrent-risk stratification PTC tumorous tissues

We collected 15 FFPE punches of PTC tumorous tissues and analyzed them by PCT-TMT-DDA proteomic pipeline.^[13]^ Out of the total 8958 identified proteins, 7850 proteins with high confidence were extracted. The proteins with missing values were further filtered, and 7171 proteins were found in all samples and used in the subsequent analysis.

**Figure 2.**
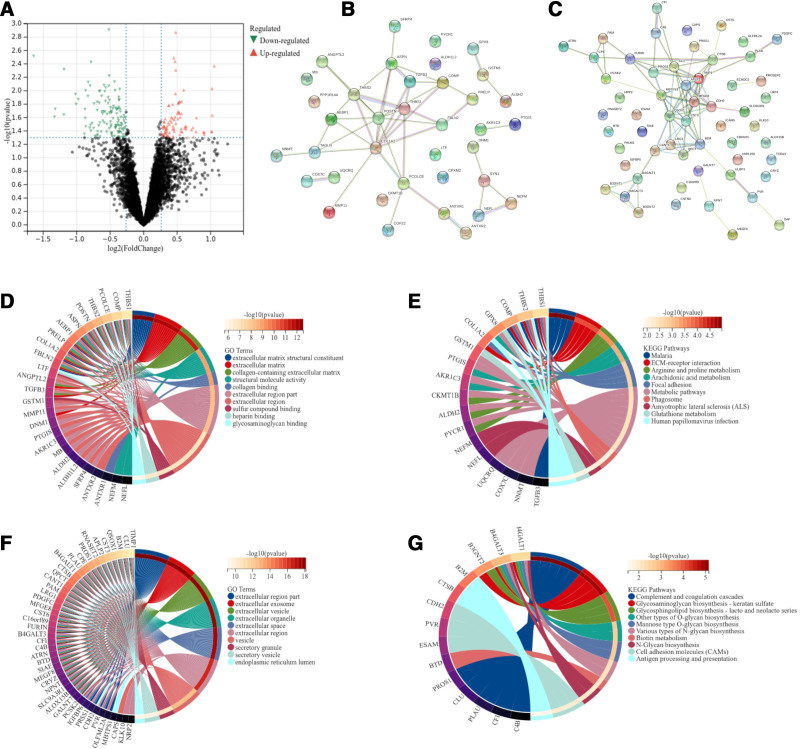
Differentially expressed proteins. (A) Volcano plot of differentially expressed proteins in tissue samples from papillary thyroid carcinoma patients with high- and low-risk stratification by the American Thyroid Association. Fold change (cutoff = 1.2, vertical lines) was plotted against the −log_10_ *P*-value (cutoff = 1.3, horizontal line). (B) Protein-protein interaction network of 38 upregulated proteins. (C) Protein-protein interaction network of 57 downregulated proteins. (D and E) The top 10 GO terms and 10 KEGG pathway enrichment of 38 upregulated proteins. (F and G) The top 10 GO terms and 10 KEGG pathway enrichments of 57 downregulated proteins. GO = Gene Ontology, KEGG = Kyoto Encyclopedia of Genes and Genomes.

We compared the expression levels of these 7171 proteins in PTC tumorous tissues with high and low recurrent risk. Using fold-change cutoff ratios of more than 1.2 for DEPs, a total of 95 differential proteins were identified, including 38 upregulated proteins and 57 downregulated proteins (Fig. 2A). STRING was used to analyze the DEPs obtained between high and low recurrence-risk PTC tumorous tissues, and a connection diagram was obtained (Fig. 2B and C). Extracellular matrix structural constituent, extracellular matrix, collagen-containing extracellular matrix, extracellular region part, and extracellular exosome were the main significantly enriched GO terms (Fig. 2D and F). Extracellular matrix-receptor interaction, arginine and proline metabolism, arachidonic acid metabolism, complement and coagulation cascades, and glycosaminoglycan biosynthesis (keratan sulfate) were the major significantly enriched KEGG terms (Figure 2E and G).

### 3.2. Bioinformatics analysis of DEPs

**Figure 3.**
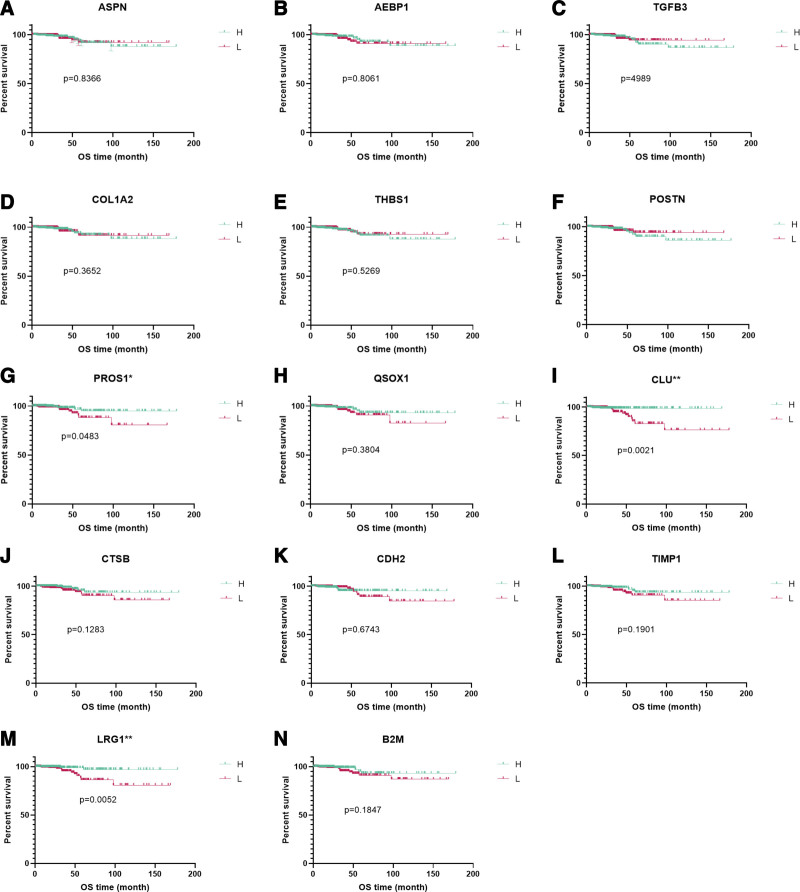
The association of mRNA expression of PROS1, CLU, LRG1, and overall survival (OS) in patients with thyroid cancer. The Cancer Genome Atlas (TCGA) dataset, which included 509 thyroid cancer tissues, provided the mRNA expression levels. There were 2 groups established from the TCGA patient samples: Those with low mRNA expression (L) (255 patients) and those with high mRNA expression (H) (254 patients). *P < .05; **P < .01. CLU = clusterin, LRG1 = leucine-rich α-2-glycoprotein 1, PROS = protein S.

An essential protein, such as one that is associated with at least 3 other proteins, is known as a hub protein. The analysis of hub protein’s mRNA expression used data from TCGA’s thyroid cancer expression dataset. Then Kaplan–Meier survival analysis was used to calculate OS based on the mRNA expression of the hub proteins. Among them, patients with high expression of PROS1, CLU, and LRG1 had better OS with *P*-value <0.05 (Fig. 3), which other proteins did not exhibit.

**Figure 4.**
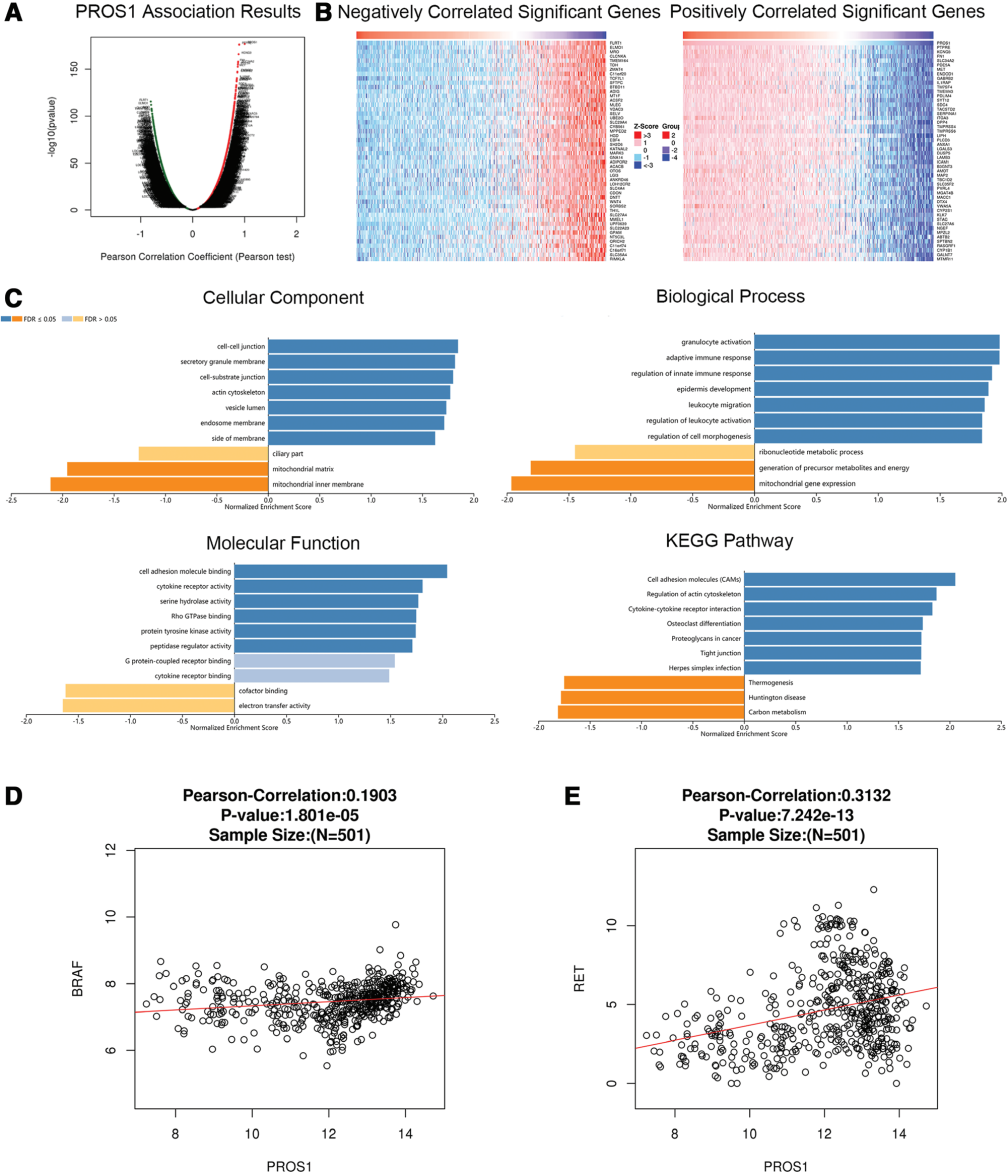
Genes differentially expressed in correlation with PROS1 in thyroid cancer (Linked Omics). (A) We used a Pearson test to look for correlations between PROS1 and genes with differential expression in thyroid cancer. (B) Heat maps showing genes negatively and positively correlated with PROS1 in thyroid cancer (top 50). Genes that are positively correlated are shown in red, while those that are negatively correlated are shown in green. (C) The significantly enriched GO annotations and KEGG pathways of PROS1 co-expression genes in thyroid cancer were analyzed using ORA, including cellular components, biological process, molecular function, and KEGG pathway. The column represents the Enrichment ratio. The scatter plot shows the Pearson correlation of PROS1 expression with expression of (D) BRAF and (E) RET. GO = Gene Ontology, KEGG = Kyoto Encyclopedia of Genes and Genomes, ORA = overrepresentation enrichment analysis, PROS = protein S.

**Figure 5.**
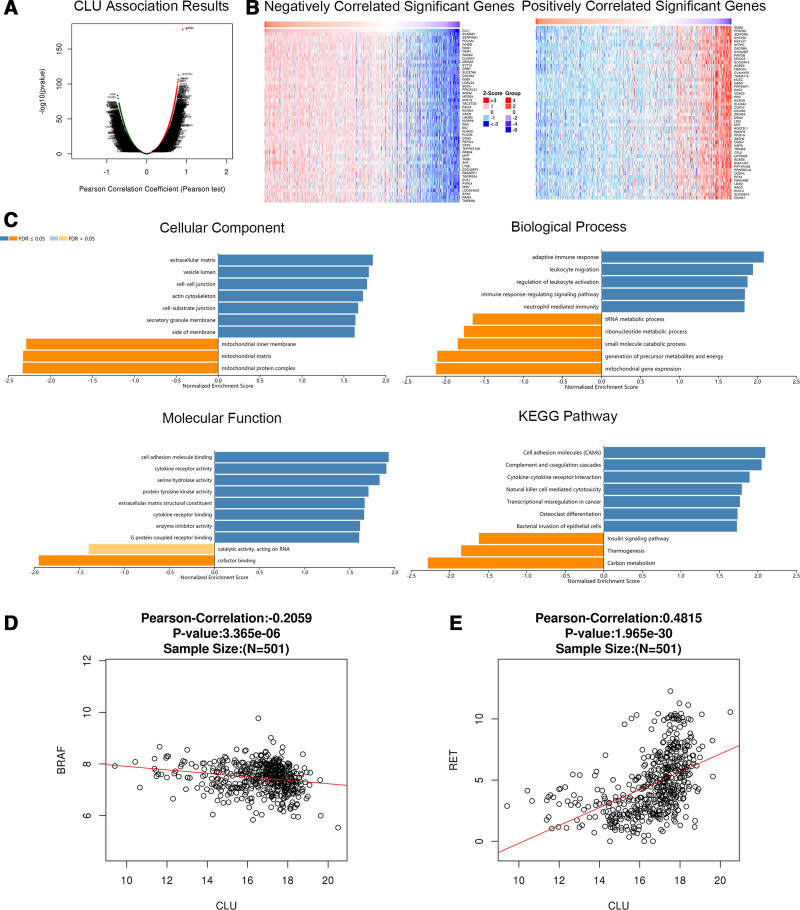
Genes differentially expressed in correlation with CLU in thyroid cancer (Linked Omics). (A) A Pearson test was used to analyze correlations between CLU and genes differentially expressed in thyroid cancer. (B) Heat maps showing genes negatively and positively correlated with CLU in thyroid cancer (top 50). Red indicates positively correlated genes, and green indicates negatively correlated genes. (C) The significantly enriched GO annotations and KEGG pathways of CLU co-expression genes in thyroid cancer were analyzed using ORA, including cellular components, biological process, molecular function, and KEGG pathway. The column represents the enrichment ratio. The scatter plot shows the Pearson correlation of CLU expression with the expression of (D) BRAF and (E) RET. CLU = clusterin, GO = Gene Ontology, KEGG = Kyoto Encyclopedia of Genes and Genomes, ORA = overrepresentation enrichment analysis.

**Figure 6.**
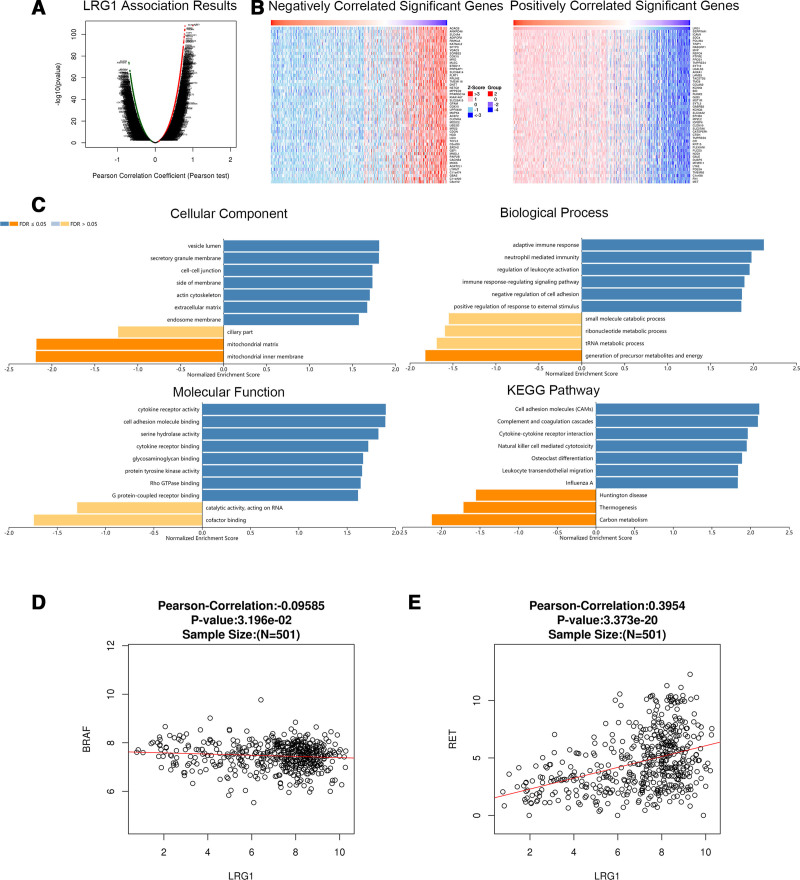
Genes differentially expressed in correlation with LRG1 in thyroid cancer (Linked Omics). (A) Correlations between LRG1 and genes differentially expressed in thyroid cancer were examined using the Pearson test. (B) Heat maps showing genes negatively and positively correlated with LRG1 in thyroid cancer (top 50). Red indicates positively correlated genes, and green indicates negatively correlated genes. (C) The significantly enriched GO annotations and KEGG pathways of LRG1 co-expression genes in thyroid cancer were analyzed using ORA, including cellular components, biological process, molecular function, and KEGG pathway. The column represents the enrichment ratio. The scatter plot shows the Pearson correlation of LRG1 expression with expression of (D) BRAF and (E) RET. GO = Gene Ontology, KEGG = Kyoto Encyclopedia of Genes and Genomes, LRG1 = leucine-rich α-2-glycoprotein 1, ORA = overrepresentation enrichment analysis.

We analyzed mRNA sequencing data from thyroid cancer patients from TCGA using the functional module of Linked Omics. Figures 4A, 5A, and 6A show the genes significantly related to PROS1, CLU, and LRG1, respectively. The heatmap shows the top 50 significant groups of genes with positive and negative correlations with PROS1, CLU, and LRG1 (Figs. 4B, 5B, and 6B). We looked for significant GO pathways using overrepresentation enrichment analysis. The results showed that the extracellular matrix, side of the membrane, and cell-cell junction were the main locations of the PROS1, CLU, and LRG1 differentially expressed genes. They were found to be more prevalent in KEGG pathway analysis and are involved in natural killer cell-mediated cytotoxicity, cell adhesion, cofactor binding, and G protein-coupled receptor binding (Figs. 4C, 5C, and 6C). Figures 4D, 5D, and 6D and Figures 4E, 5E, and 6E show the correlation between PROS1, CLU, and LRG1 and BRAF and RET genes, respectively. The correlation between CLU, BRAF, and RET is −0.2059 and 0.4818, respectively, which is significant.

We assessed 8 clinicopathological characteristics of patients using clinical data from our center. We statistically analyzed the relationships between recurrence risk, BRAF gene mutation, gender, age at first thyroid cancer diagnosis, tumor size, extrathyroidal invasion, lymph node metastasis, and distant metastasis as well as the expression levels of PROS1, CLU, and LRG1. The findings demonstrated that there was no connection between these clinical characteristics in PTC and the level of PROS1 expression (Table 1). The risk of recurrence, extrathyroidal invasion, and lymph node metastasis were all significantly correlated with the level of CLU expression (*P* = .021, .021, and .004, respectively) (Table 2). There was no observable relationship between the level of LRG1 expression and these clinical traits (Table 3).

**Table 1:** Correlation between PROS1 protein expression and clinicopathological parameters of thyroid cancer patients.

Parameter	PROS1 expression			χ^2^	*P*-value
	Number	High	Low		
Recurrent					
High	7	2	5		
Low	8	6	2	3.233	0.201
BRAF					
N	7	4	3		
P	8	4	4	0.077	1
Gender					
Female	11	6	5		
Male	4	2	2	0.024	1
Age					
<55	8	6	2		
≥55	7	2	5	3.233	0.201
Tumor diameter					
≤1	8	5	3		
>1	7	3	4	0.579	0.809
Extra invasion					
No	8	6	2		
Yes	7	2	5	3.233	0.201
LN metastasis					
No	9	7	2		
Yes	6	1	5	5.402	0.073
Metastasis					
No	13	8	5		
Yes	2	0	2	2.637	0.388

LN = lymph node, PROS1 = protein S.

*P* < .05 was considered to have statistical significance.

**Table 2:** Correlation between CLU protein expression and clinicopathological parameters of thyroid cancer patients.

Parameter	CLU expression			χ^2^	*P*-value
	Number	High	Low		
Recurrent					
High	7	1	6		
Low	8	7	1	8.040	0.021
BRAF					
N	7	3	4		
P	8	5	3	0.579	0.0809
Gender					
Female	11	6	5		
Male	4	2	2	0.024	1
Age					
<55	8	6	2		
≥55	7	2	5	3.233	0.201
Tumor diameter					
≤1	8	6	2		
>1	7	2	5	3.233	0.201
Extra invasion					
No	8	7	1		
Yes	7	1	6	8.040	0.021
LN metastasis					
No	9	8	1		
Yes	6	0	6	11.429	0.004
Metastasis					
No	13	8	5		
Yes	2	0	2	2.637	0.388

CLU = clusterin, LN = lymph node.

*P* < .05 was considered to have statistical significance.

**Table 3:** Correlation between LRG1 protein expression and clinicopathological parameters of thyroid cancer patients.

Parameter	LRG1 expression			χ^2^	*P*-value
	Number	High	Low		
Recurrent					
High	7	3	4		
Low	8	5	3	0.579	0.0809
BRAF					
N	7	5	2		
P	8	3	5	1.727	0.426
Gender					
Female	11	5	6		
Male	4	3	1	1.029	0.668
Age					
<55	8	5	3		
≥55	7	3	4	0.579	0.809
Tumor diameter					
≤1	8	5	3		
>1	7	3	4	0.579	0.809
Extra invasion					
No	8	5	3		
Yes	7	3	4	0.579	0.809
LN metastasis					
No	9	5	4		
Yes	6	3	3	0.045	1
Metastasis					
No	13	7	6		
Yes	2	1	1	0.010	1

LN = lymph node, LRG1 = leucine-rich α-2-glycoprotein 1.

*P* < .05 was considered to have statistical significance.

### 3.3. Verification of candidate biomarkers by IHC

**Figure 7.**
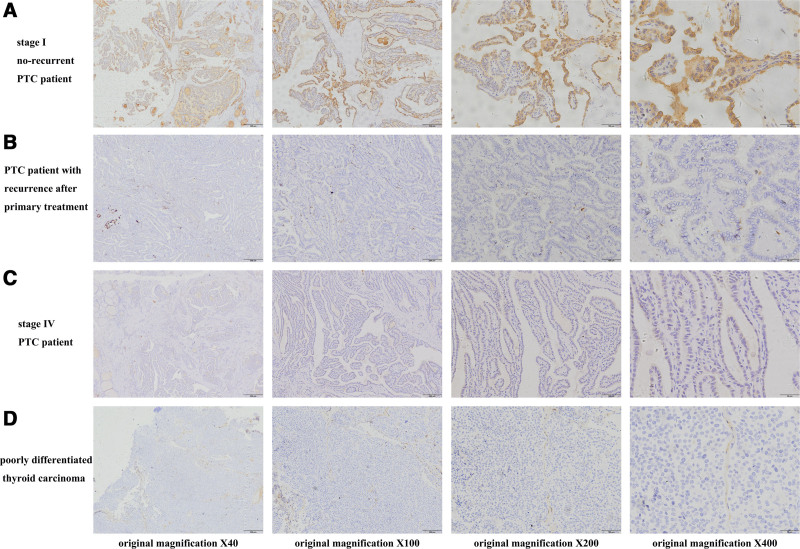
Representative immunohistochemical staining of PROS1 in different PTC tissues. (A) Stage I (AJCC) without recurrence PTC tissue shows intense staining for PROS1. (B) PTC tissue involved with tumor recurrence after primary surgical treatment shows low staining for PROS1. (C) Stage IV (AJCC) PTC tissue shows low staining for PROS1. (D) Tumor cells of poorly differentiated thyroid carcinoma show low staining for PROS1. AJCC = American Joint Committee on Cancer 8th, PROS = protein S, PTC = papillary thyroid carcinoma.

**Figure 8.**
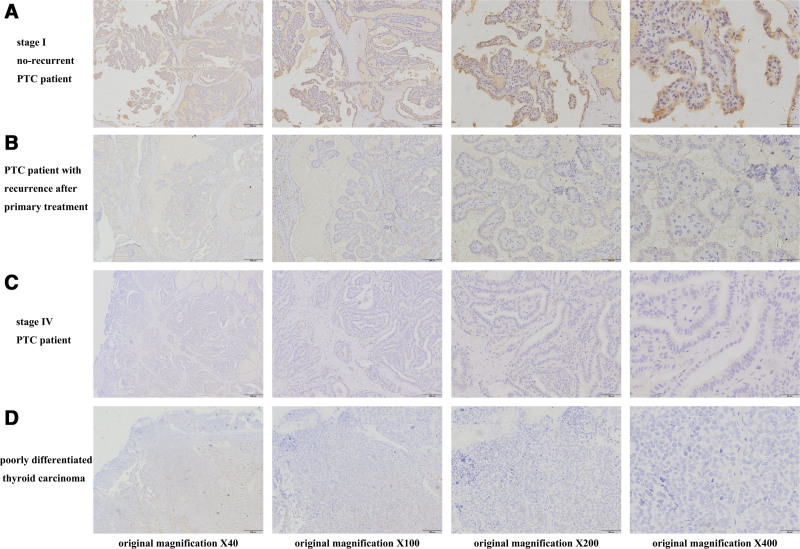
Representative immunohistochemical staining of CLU in different PTC tissues. (A) Stage I (AJCC) without recurrence PTC tissue shows intense staining for CLU. (B) PTC tissue involved with tumor recurrence after primary surgical treatment shows low or moderate staining for CLU. (C) Stage IV (AJCC) PTC tissue shows low or moderate staining for CLU. (D) Tumor cells of poorly differentiated thyroid carcinoma show low staining for CLU. AJCC = American Joint Committee on Cancer 8th, CLU = clusterin, PTC = papillary thyroid carcinoma.

**Figure 9.**
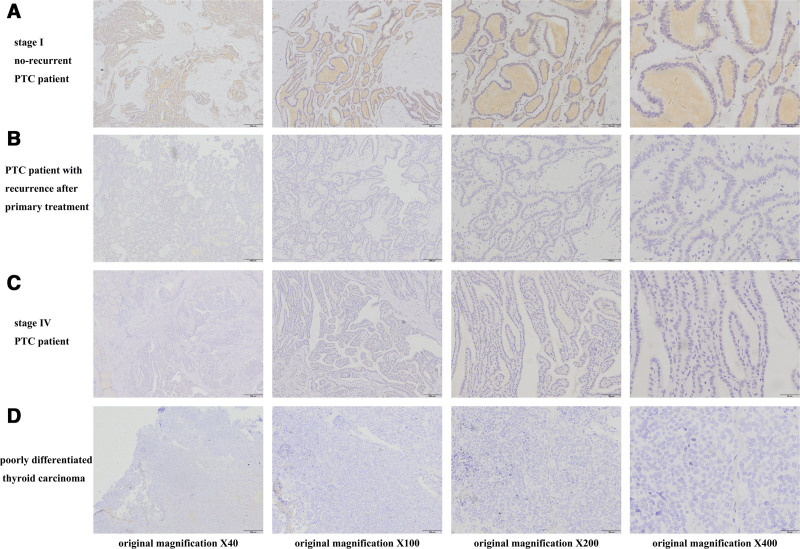
Representative immunohistochemical staining of LRG1 in different PTC tissues. (A) Stage I (AJCC) without recurrence PTC tissue shows intense staining for LRG1. (B) PTC tissue involved with tumor recurrence after primary surgical treatment shows low staining for LRG1. (C) Stage IV (AJCC) PTC tissue shows low staining for LRG1. (D) Tumor cells of poorly differentiated thyroid carcinoma show low staining for LRG1. AJCC = American Joint Committee on Cancer 8th, LRG1 = leucine-rich α-2-glycoprotein 1, PTC = papillary thyroid carcinoma.

In 53 cases of PTC specimens, IHC was used to confirm the expression levels of the potential prognostic biomarkers PROS1, CLU, and LRG1. Figures 7A, 8A, and 9A show that stage I (American Joint Committee on Cancer 8th, AJCC 8th) PTC patients with no recurrence typically had intense PROS1, CLU, and LRG1 staining, whereas PTC patients with tumor recurrence after primary surgical treatment typically had low or moderate staining (Figs. 7B, 8B, and 9B). Additionally, poorly differentiated thyroid carcinoma and PTC tumor cells at stage IV (AJCC 8th) PTC typically expressed low to moderate levels of PROS1, CLU, and LRG1 (Figs. 7C, 8C, and 9C and Figs. 7D, 8D, and 9D).

### 3.4. Statistical analysis of immunohistochemical data

Additionally, we investigated the relationship between the clinicopathological traits and the downregulation of PROS1, CLU, and LRG1 in PTC tumorous tissues using the nonparametric Mann–Whitney *U* test. Inverse correlations were found between the IHC scores of PROS1 (*P* = .024), CLU (*P* = .010), and LRG1 (*P* = .038) and PTC patients with higher tumor classification, respectively (Tables 4–6). Furthermore, PTC patients with advanced AJCC stage had significantly lower PROS1, CLU, and LRG1 IHC scores than PTC patients in early-stage disease (*P* = .005, *P* = .012, and *P* = .039, respectively) (Tables 4–6). PTC patients with lymph node metastasis showed significantly lower IHC scores for PROS1 (*P* < .001), CLU (*P* = .003), and LRG1 (*P* < .001) than patients without metastasis, respectively (Tables 4–6). Additionally, PTC patients with tumor extrathyroidal extension had significantly lower PROS1, CLU, and LRG1 IHC scores compared to patients without extrathyroidal extension (*P* = .001, *P* = .001, and *P* = .001, respectively) (Tables 4–6). Multiple PTC patients showed significantly lower IHC scores for PROS1 (*P* = .004), CLU (*P* = .040), and LRG1 (*P* = .006) than solitary patients, respectively (Tables 4–6). The expression levels of PROS1 (*P* = .001), CLU (*P* < .001), and LRG1 (*P* = .002) were significantly lower in PTC patients with large tumor size (>2 cm) than in small tumor size patients (≤2 cm), respectively (Tables 4–6). The expression levels of PROS1, CLU, and LRG1 had no association with the patient’s age and sex in PTC. These results indicate that low or moderate expression of PROS1, CLU, and LRG1 in PTC tumor cells was significantly associated with poor clinicopathological characteristics.

**Table 4:** Correlation of PROS1 protein expression with clinicopathological characteristics.

Characteristics	Score, n	Low (0–2)	Moderate (3–4)	High (5–6)	*P* value*
Age (yr)					
<55	39	17	5	17	
≥55	14	8	2	4	.330
Gender					
Male	14	7	1	6	
Female	39	18	6	15	1.000
Extrathyroidal extension					
No	28	6	5	17	
Yes	25	19	2	4	<.001
Multifocality					
Solitary	25	7	3	15	
Multiple	28	18	4	6	.004
Tumor sizes (cm)					
≤2	35	11	5	19	
>2	18	14	2	2	.001
Tumor classification					
T1 + T2	38	14	6	18	
T3 + T4	15	11	1	3	.024
Lymph node classification					
N0	15	1	3	11	
N1	38	24	4	10	<.001
AJCC stage					
I + II	46	18	7	21	
III + IV	7	7	0	0	.005

AJCC = American Joint Committee on Cancer 8th, PROS = protein S.

* *P* value represents the probability from the nonparametric Mann–Whitney *U* test; *P* < .05 was considered to have statistical significance.

**Table 5:** Correlation of CLU protein expression with clinicopathological characteristics.

Characteristics	Score, n	Low (0–2)	Moderate (3–4)	High (5–6)	*P* value*
Age (yr)					
<55	39	6	8	25	
≥55	14	4	4	6	.157
Gender					
Male	14	2	1	11	
Female	39	8	11	20	.124
Extrathyroidal extension					
No	28	3	2	23	
Yes	25	7	10	8	.001
Multifocality					
Solitary	25	4	2	19	
Multiple	28	6	10	12	.040
Tumor sizes (cm)					
≤2	35	3	4	28	
>2	18	7	8	3	<.001
Tumor classification					
T1 + T2	38	6	5	27	
T3 + T4	15	4	7	4	.010
Lymph node classification					
N0	15	1	0	14	
N1	38	9	12	17	.003
AJCC stage					
I + II	46	7	9	30	
III + IV	7	3	3	1	.012

AJCC = American Joint Committee on Cancer 8th, CLU = clusterin.

* *P* value represents the probability from the nonparametric Mann–Whitney *U* test; *P* < .05 was considered to have statistical significance.

**Table 6:** Correlation of LRG1 protein expression with clinicopathological characteristics.

Characteristics	Score, n	Low (0–2)	Moderate (3–4)	High (5–6)	*P* value*
Age (yr)					
<55	39	20	5	14	
≥55	14	8	1	5	.813
Gender					
Male	14	8	1	5	
Female	39	20	5	14	.813
Extrathyroidal extension					
No	28	9	3	16	
Yes	25	19	3	3	.001
Multifocality					
Solitary	25	8	4	13	
Multiple	28	20	2	6	.006
Tumor sizes (cm)					
≤2	35	14	3	18	
>2	18	14	3	1	.002
Tumor classification					
T1 + T2	38	17	4	17	
T3 + T4	15	11	2	2	.038
Lymph node classification					
N0	15	2	3	10	
N1	38	26	3	9	<.001
AJCC stage					
I + II	46	22	5	19	
III + IV	7	6	1	0	.039

AJCC = American Joint Committee on Cancer 8th, LRG1 = leucine-rich α-2-glycoprotein 1.

* *P* value represents the probability from the nonparametric Mann–Whitney *U* test; *P* < .05 was considered to have statistical significance.

**Figure 10.**
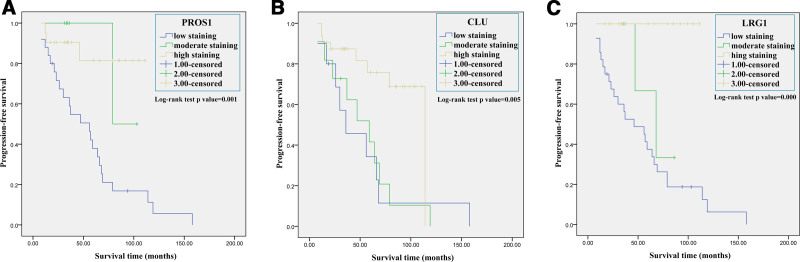
Cumulative progression-free survival (PFS) curves of papillary thyroid carcinoma patients. Patients were divided into groups based on their immunohistochemistry scores for PROS1, CLU, and LRG1. (A) Kaplan–Meier method (log-rank analysis) showed patients with low PROS1 expression had worse PFS than patients with moderate or high expression. (B) Kaplan–Meier method (log-rank analysis) showed patients with low or moderate CLU expression had worse PFS than patients with high expression. (C) Kaplan–Meier method (log-rank analysis) showed patients with low or moderate LRG1 expression had worse PFS than patients with high expression. CLU = clusterin, LRG1 = leucine-rich α-2-glycoprotein 1, PROS = protein S.

**Figure 11.**
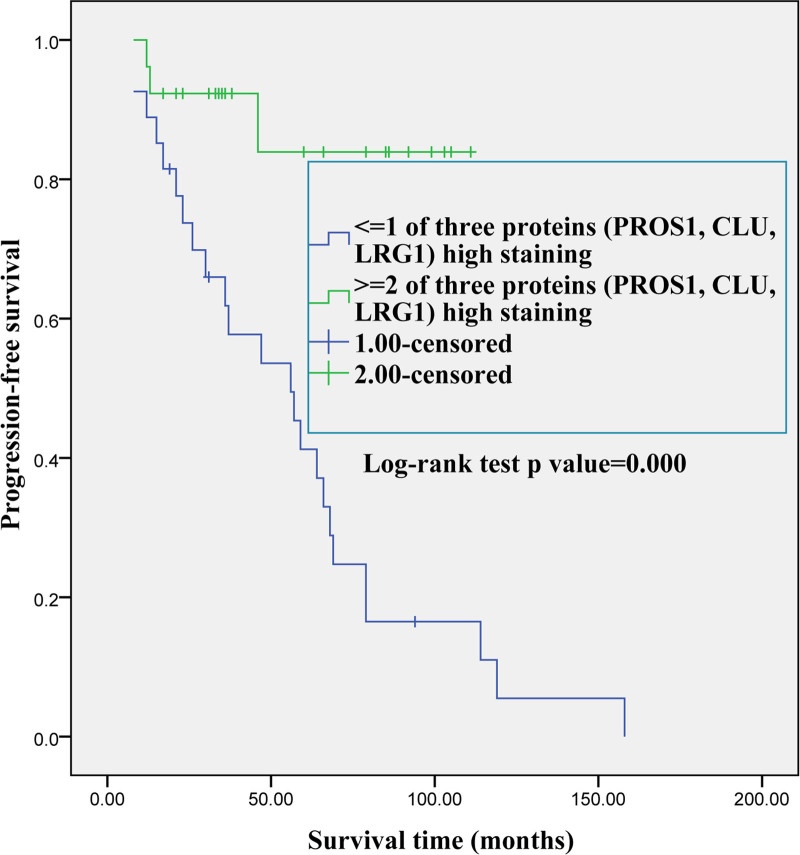
Patient’s cumulative progression-free survival (PFS) curves for papillary thyroid carcinoma (PTC) based on PROS, CLU, and LRG1 immunohistochemistry levels. Kaplan–Meier method (log-rank analysis) showed PTC patients with 2 or 3 PROS1, CLU, or LRG1 proteins high expression had better PFS than patients with zero or one of PROS1, CLU, or LRG1 proteins high expression. CLU = clusterin, LRG1 = leucine-rich α-2-glycoprotein 1, PROS = protein S.

We assessed the prognostic relevance of PROS1, CLU, and LRG1 in PTC. Kaplan–Meier analysis indicated that patients with low PROS1 expression in tumorous tissue had worse PFS than patients with moderate or high expression (*P* = .001; Fig. 10A). Furthermore, patients with high CLU (*P* = .005) or LRG1 (*P* = .000) expression had better PFS than patients with low or moderate CLU or LRG1 expression (Fig. 10B and C). In addition, we found that PTC patients with 2 or 3 PROS1, CLU, or LRG1 proteins high expression had better PFS than those with zero or one of PROS1, CLU, or LRG1 proteins high expression (Fig. 11).

## 4. Discussion

In the present study, we adopted PCT-TMT-DDA proteomic pipeline to identify DEPs between high and low recurrent-risk PTC tumorous tissues. A total of 7171 proteins were found in PTC tumorous cells, and 95 DEPs, including 38 upregulated proteins and 57 downregulated proteins, were found between high and low recurrent-risk groups. These results provided significant new understandings of the causes and prognosis of PTC. A preprint has previously been published and the present paper is modified from the preprint.^[14]^

The wide range of tumor behaviors poses problems for clinical risk assessment and PTC management decision-making. Present-day PTC treatments range from active surveillance to total thyroidectomy combined with RAI therapy.^[7]^ The best approach for treating a PTC patient will depend on whether they have a good or poor prognosis. In this study, we have identified 3 novel prognosis protein biomarkers of PTC-PROS1, CLU, and LRG1. GO enrichment analysis suggested that the 3 proteins may play a role in tumorigenesis through biological regulation, metabolic processes, and binding-related mechanisms. Additionally, KEGG pathway analysis showed that the 3 proteins were mainly concentrated in cancer-related pathways like focal adhesion pathways and proteoglycan pathways.

The tumor-associated macrophages receptor-ligand complex’s PROS1 is a cognate ligand for it. The abnormally high expression of PROS1 played critical roles in the promotion of the growth of glioblastoma,^[15]^ oral squamous cell carcinoma,^[16]^ and colorectal cancer.^[17]^ However, in this study, we found low expression of PROS1 mRNA was significantly associated with poor OS in PTC patients. Poor clinicopathologic outcomes of PTC, such as extrathyroidal extension, multifocality, larger tumor size, advanced tumor classification, lymph node metastasis, and advanced AJCC tumor, node, metastasis (staging system for cancer) stage, were strongly associated with low PROS1 expression. PTC patients with low PROS1 expression had a worse PFS than PTC patients with moderate or high PROS1 expression. It is necessary to conduct additional in vitro and in vivo testing to confirm whether there are distinct molecular mechanisms underlying PROS1’s functions in various cancers.

Zelentsova-Levytskyi et al have revealed that PROS1 is a negative regulator of neural stem and progenitor cells (NSPCs) self-renewal through Bmi-1 signaling. Self-renewing divisions of NSPCs are significantly enhanced in the absence of PROS1, resulting in increased NSPC numbers.^[18]^ In carcinoma cells, whether PROS1 has a similar function demand further investigation. PROS1 gene expression was found to be higher in PTC samples than in healthy thyroid tissue, according to Wang et al. Although the PROS1 gene expression was statistically unrelated to the patient’s age, gender, tumor classification, extrathyroidal invasion, and *BRAFV*^*600E*^ mutation, it was strongly correlated with the classification of lymph nodes.^[19]^ In Wang et al’s study, with no patients at stages III and IV and only a few patients at stage II, our study may produce results that are incongruent.

CLU is a single-copy gene located in chromosome 8 with 9 exons in humans.^[20]^ Through alternative splicing and translation of CLU, it generates 3 isoforms: secreted (sCLU), nuclear (nCLU), and cytoplasmic (cCLU).^[21]^ CLU demonstrates isoform-specific biological functions by distinct cell localization and various protein structures.^[22]^ sCLU is overexpressed in breast, prostate, and colon cancers to prevent cell death and advance the disease.^[23–25]^ In contrast to the cytoprotective function of sCLU in the majority of cancers, nCLU demonstrates the cytotoxic property that promotes cell death in some particular cancers, and the anticancer activity of nCLU is primarily reflected in promoting cell death, inhibiting cell proliferation, and blocking metastasis and invasion.^[26]^ nCLU is defined as a tumor suppressor and functions as a proapoptotic protein in MCF-7 breast cancer cells. Relevant studies have demonstrated that in breast cancer, nCLU binds to cytosolic Ku70 and causes X-ray-induced cell death.^[27,28]^ Additionally, Kim et al found that the binding of nCLU with Bcl-XL and perhaps other antiapoptotic proteins, which led to the release and activation of Bax, was responsible for nCLU overexpression causing significant apoptotic cell death.^[29]^

In this study, we found a significant relationship between low or moderate CLU expression in PTC tissues and high-risk clinicopathologic outcomes, such as tumor extrathyroidal extension, multifocality, larger tumor size, advanced tumor classification, lymph node metastasis, and advanced AJCC tumor, node, metastasis stage. Bioinformatics analysis and IHC study of our clinical cases both showed PTC patients with higher CLU expression had a better prognosis than those with low or moderate CLU expression. In PTC, there is a significant association between BRAF mutation and extrathyroidal extension, lymph node metastasis, advanced tumor stage, disease recurrence, and even patient mortality.^[30]^ In bioinformatics analysis, we also found the expression level of CLU has a significantly negative correlation with BRAF mutation in thyroid cancer. Though we did not investigate the isoforms of CLU in this study, our results show CLU has an inhibition function in PTC, and PTC patients with low CLU expression have a poor prognosis.

The leucine-rich repeat family of proteins, including LRG1, are involved in protein-protein interactions, cell signal transduction, cell adhesion, DNA repair, immune response, and other processes.^[31]^ In several types of carcinomas, including head and neck squamous cell carcinoma, esophageal squamous cell carcinoma, and Lewis lung carcinoma cell lines, the expression levels of LRG1 were discovered to be downregulated, where it inhibits tumor cell proliferation, migration, and invasion.^[32–35]^ Takemoto et al reported that the high expression of LRG1 inhibited the growth of Lewis lung carcinoma cells through the TGFβ1-induced apoptosis pathway.^[34]^ Wen et al clarified that LRG1 is an independent prognostic factor in endometrial carcinoma patients. Patients with stages III and IV endometrial carcinomas had a higher rate of LRG1-negative tumors than those with stages I and II. The OS of the LRG1-positive group was significantly better than that of the LRG1-negative group.^[36]^ Although LRG1 did not affect cell proliferation, it was found that hepatocellular carcinoma (HCC) had lower levels of LRG1 expression. The migratory and invasive potential of HCC cells was reduced by ectopic overexpression of LRG1, whereas silencing LRG1 could enhance migration and invasion of HCC cells.^[35]^

In this study, we also found that LRG1-low or moderate expression rate was higher in stages III and IV PTC patients than in stages I and II. Poor clinicopathological outcomes of PTC were significantly associated with LRG1-low or moderate expression. PFS was significantly better in the LRG1-high expression group than in the LRG1-low or moderate expression groups. In bioinformatics analysis, there was a negative correlation between LRG1 expression and *BRAF* mutation, but it was not significant. PTC patients with low or moderate LRG1 expression had a worse prognosis than those with high expression.

However, the expression, impact, and potential mechanisms of LRG1 on malignant carcinomas remain controversial. Many other cancers have high levels of LRG1, which promotes tumor cell proliferation and invasion.^[37]^ Further studies are warranted to address such discrepancies.

The PTC PFS of patients with 2 or 3 of the novel prognostic proteins (PROS1, CLU, and LRG1) high expression was significantly better than that of patients with only one or no of the proteins (PROS1, CLU, and LRG1) high expression, suggesting an additive and synergistic effect of the coexisting 3 proteins low or moderate expression. The protein-protein interaction network of proteins that were downregulated also revealed a strong correlation between the aforementioned 3 proteins.

The current study, however, has several limitations. First, a retrospective cohort study has some inherent characteristics. Second, there were not many PTC patients included in the IHC study. It is necessary to conduct large sample prospective studies to confirm the usefulness of novel prognostic protein biomarkers in clinical settings. Finally, in this study, we did not investigate the impact of the novel prognostic biomarkers on tumorigenesis and progression.

## 5. Conclusions

Proteomic analysis was used in the current study to identify significant differences between the protein expression profiles of PTC patients with high and low recurrent risk. Bioinformatics analysis and IHC study show low or moderate expression of PROS1, CLU, or LRG1 was associated with poor prognosis in PTC. Thus, PROS1, CLU, and LRG1 potentially offer clinical value in directing personal treatment for PTC patients.

## Acknowledgments

We thank Tiannan Guo, Yaoting Sun, and Lu Li from Westlake University for helping with proteomic sample preparation and data acquisition. We also thank for assistance in peptide fractionation by the Mass Spectrometry & Metabolomics Core Facility at the Center for Biomedical Research Core Facilities of Westlake University.

## Author contributions

**Conceptualization:** Haili Sun, Kehao Le.

**Data curation:** Haili Sun, Jianbiao Wang, Nizhen Xu, Kehao Le.

**Formal analysis:** Haili Sun, Jianbiao Wang, Nizhen Xu, Kehao Le.

**Funding acquisition:** Jianbiao Wang.

**Investigation:** Kehao Le.

**Methodology:** Haili Sun, Jianbiao Wang, Nizhen Xu, Kehao Le.

**Project administration:** Haili Sun, Jianbiao Wang, Kehao Le.

**Resources:** Haili Sun, Nizhen Xu, Kehao Le.

**Software:** Kehao Le.

**Supervision:** Kehao Le.

**Validation:** Kehao Le.

**Writing – original draft:** Haili Sun, Jianbiao Wang, Kehao Le.

**Writing – review & editing:** Haili Sun, Kehao Le.
